# Job insecurity, psychological safety, work alienation, and anxiety in the hotel industry: a moderated-mediated analyses

**DOI:** 10.3389/fpsyg.2025.1615426

**Published:** 2025-08-13

**Authors:** Hassane Gharbi, Abu Elnasr E. Sobaih, Nadir Aliane

**Affiliations:** ^1^Management Department, Faculté des Sciences Économiques et de Gestion de Nabeul, Université de Carthage, Tunis, Tunisia; ^2^Management Department, College of Business Administration, King Faisal University, Al-Ahsa, Saudi Arabia

**Keywords:** hotels, job insecurity (JI), psychological safety (PS), work anxiety (AX), work alienation (AL)

## Abstract

**Introduction:**

When hotels no longer keep their promises to their employees, particularly in terms of job security, employees may feel less motivated to perform their tasks. With the certainty of being wronged, they risk falling prey to the hegemony of workplace alienation and anxiety.

**Methods:**

For this purpose, a quantitative methodology, particularly structural equation modeling, was employed to analyse responses from 516 employees working in hotels in Tunisia.

**Results and discussion:**

The findings confirmed that job insecurity had a significant and positive influence on anxiety and alienation at work. In addition, alienation at work significantly and positively influenced the anxiety experienced by employees. The results showed that the relationship between job insecurity and anxiety remained significant after the introduction of work alienation as a mediating variable. The mediation effect of work alienation was confirmed to be partial in the relationship between job insecurity and work-related anxiety. The moderator effect analysis revealed that psychological safety significantly reduced the negative impact of job insecurity on employees' anxiety levels. The research provides useful suggestions to help managers and decision-makers mitigate feelings of alienation at work and high levels of anxiety among employees.

## 1 Introduction

Hotels face many challenges in retaining their human resources (Aliane and Gharbi, 2023). For this reason, a balanced social atmosphere and a healthy working environment (Gharbi et al., 2023) have become more essential than ever, as they now represent a major challenge and an imperative that no company can ignore. In their research, Hitchner et al. (2023) argued that promoting conditions conducive to work can direct employees' efforts toward the achievement of common goals. Psychological safety is a highly interesting aspect, as it reflects the mental health of employees, enabling them to make decisions freely (Gharbi et al., 2023). In addition, psychological safety has a significant impact on the reduction of turnover intention among hotel employees (Sobaih et al., 2022).

In the same vein, Conservation of Resources Theory (CRT) (Hobfoll, 1989) posits that employees are strongly motivated to find a physical context that ensures their success, wellbeing and personal satisfaction. CRT suggests that, by nature, human beings continually strive to maintain and develop their personal, social and material resources. Hobfoll (2011) described these resources as concepts that are either naturally valued, such as health and inner peace, or as means to an end, such as money and recognition. These resources are driven by intrinsic value for individuals, since they define them as such—notably in terms of personal value and self-respect (Sobaih et al., 2024). On the other hand, it should be noted that the risk of dispossessing these resources is significantly more serious than their possession, since, although they are difficult to acquire, they are exceedingly easy to lose. It goes without saying that when these resources are threatened or at risk of being lost, affected individuals are likely to endure severe stress (Hobfoll, 1998) and a vulnerability that can lead them to experience unprecedented anxiety (Abouelenien et al., 2024).

On this subject, Windayanti et al. (2025) argued that job insecurity is a recurring feeling of anxiety at work. The destructive influence of job insecurity affects both material and psychological aspects, such as the emergence of a feeling of dissatisfaction (Saputra et al., 2020), the culture of social loafing (Edrees et al., 2023), the intentional decline in productivity (Dewi and Prahara, 2025), the appearance of the intention to leave one's job (Horpynich et al., 2025), anxiety (Khudaykulov et al., 2024), social alienation (Gharbi and Sobaih, 2023), alienation at work (Abouelenien et al., 2024; Mahmoud et al., 2024), and anger (Gahrmann et al., 2024).

While earlier studies (e.g., Sandhu and Fatima, 2021; Mahmoud et al., 2024) found that work alienation is an outcome of work anxiety, the current research assumes that perceived job insecurity could significantly affect both work alienation and anxiety. This is because, like other sectors, when hotels are no longer able to keep their promises to their employees, particularly in terms of job insecurity, the latter may feel less motivated to perform their tasks (Aliedan et al., 2022; Sobaih et al., 2024). Tormented by a feeling of discomfort, employees, certain that they are being wronged, may develop withdrawal behaviors and, in extreme cases, be tempted to leave their companies, since their mental and physical wellbeing is being undermined (Alyahya et al., 2021; Aliedan et al., 2022). In this uncertain landscape that characterizes their daily lives, they become easy prey to the hegemony of stress, alienated work and anxiety (Hobfoll, 1998, 2011). This research aims to turn the spotlight on two constructs of crucial importance. The first, alienation at work, is proposed to act as a mediator in the relationship between job insecurity and anxiety. To the best of the research team's knowledge, this is the first study to investigate this mediation. The second focus is on the role of a moderating variable, namely psychological safety. This research addresses these two understudied issues by measuring the mediation effect of work alienation and the moderation effect of psychological safety, as well as their potential to alleviate the influence of job insecurity on work anxiety.

The objectives of this research are to assess the direct impact of job insecurity on employee anxiety; measure the indirect impact of job insecurity on employee anxiety, particularly through work alienation; discern the type of mediation (partial or total) of work alienation, if any; and determine whether psychological safety can moderate the influence of job security on employee anxiety. The research questions are as follows: in what way can alienation at work play a mediating role in the relationship between job insecurity and employee anxiety? In what way can psychological safety play a moderating role in the relationship between job insecurity and employee anxiety?

## 2 Theoretical framework and operationalisation of hypotheses

### 2.1 Job insecurity and work anxiety

Due to seasonal fluctuations, job insecurity remains a ghost that haunts employees in the hospitality industry (Darvishmotevali, 2025). This insecurity is known as the fear of losing one's job for reasons that are sometimes beyond the control of the individual in question (Shoss, 2017; Singh et al., 2025). Hence, this research defines it as the powerlessness felt in maintaining one's job. Its occurrence can have negative repercussions on the employee's physical and psychological resources (Mauno et al., 2007). Indeed, job insecurity is likely to be detrimental to employees' wellbeing (Haar et al., 2025).

Considering wellbeing at work, Lu et al. (2019) stated that workplaces characterized by psychological safety are synonymous with better employee wellbeing. Hence, the risk of running up against anxiety cannot be overlooked, which is an iterative mental health problem (Wood et al., 2025), especially when it comes to job insecurity. This problem is even more serious since redundant anxiety can generate depressive states in some cases (Ernst et al., 2021). Work anxiety refers to a range of feelings such as tension, nervousness and discomfort associated with the performance of a task (Cheng and McCarthy, 2018). Anxiety at work often appears to be related to job insecurity, as it is rooted in concerns about uncertainty or potential danger, which characterize the latter (Miceli and Castelfranchi, 2005).

Numerous studies have revealed that job insecurity has a positive impact on anxiety outcomes. For example, Boya et al. (2008) conducted a cross-sectional study across 11 hospitals to examine the effects of job insecurity on the anxiety levels of 462 nurses working in healthcare in Izmir, Turkey. The study observed that job insecurity significantly and positively affected the nurses' anxiety (Boya et al., 2008). In England, where job insecurity has increased, a survey conducted by the Workplace Employment Relations Survey (WERS) in 2011, involving over 16,000 employees across 1,100 organizations, confirmed that job insecurity is associated with work-related anxiety (Wang et al., 2020). Llosa et al. (2018) conducted research in Spain based on a systematic meta-analysis of 56 independent samples, totalling 53,405 participants. The results revealed a relationship between job insecurity and mental health. In other words, job insecurity is linked to anxiety, emotional burnout and low general satisfaction with working life. Recently, An et al. (2023) conducted a study on white-collar employees working in many organizations and institutions in the United States, which found that employees who experienced stress due to job insecurity were likely to develop acute symptoms of anxiety. Based on the above information, the following hypothesis was formulated:

*H1: Job insecurity has a positive impact on the anxiety experienced by hotel employees*

### 2.2 Job insecurity and work alienation

Job insecurity refers to the anxiety experienced by an employee regarding a potential threat, typically an involuntary event, that may prevent them from keeping their current job. Job insecurity can stem from a range of factors, such as the erratic state of the economy, the need to relocate, the rise of technological progress and the pervasiveness of artificial intelligence—all of which can render certain organizational tasks obsolete (Lee et al., 2018). Idris et al. (2023); Nguyen P. T. et al. (2025) established a typology of job insecurity. Firstly, they identified a quantitative type of job insecurity, known as the risk of job loss. Secondly, they outlined a qualitative type of insecurity, identified as the perception of a potential threat in relation to important specificities of the job in question.

A meta-analysis conducted by Sverke et al. (2002) showed that job insecurity has a negative impact on job quality, particularly the wellbeing of employees, through a reduction in both physical and mental health. Furthermore, the results of research carried out in Egypt indicated a negative and statistically significant relationship between nurses' perception of job security and their experience of alienation at work (Badran and Khaled, 2021). Similarly, Zaki and Al-Romeedy (2018), in a study conducted with 229 employees in Egyptian travel agencies and the tourism industry, found a significant negative relationship between job security and work alienation. Therefore, it could be concluded based on these two studies that an experience of job insecurity will lead to a feeling of alienation at work. Moreover, Abouelenien et al. (2024), based on 421 valid responses from full-time employees in Egyptian category A travel agencies and five-star hotels, found that job insecurity has a positive influence on work alienation.

Many studies (Chiaburu et al., 2014; Tummers et al., 2015; Usman et al., 2020) have found that alienation at work reflects badly on individuals and the organizations they work for. The devaluation and disrespect of employees as human beings often appear to be synonymous with alienation at work (Liu et al., 2025). According to Chiaburu et al. (2013), alienation at work is a concept that refers to a psychological state of isolation from oneself and one's relationships with others, both within and outside the work context. Based on this, the following hypothesis was proposed:

*H2: Job insecurity has a positive impact on feelings of work alienation among hotel employees*.

### 2.3 Work alienation and work anxiety

From a socio-professional perspective, alienation at work could be defined as a feeling of disunity and indolence that emerges among employees when they can no longer find fulfillment in their workplace (Chiaburu et al., 2013). Immediately, a range of feelings such as tension, nervousness and discomfort related to work performance emerges. Anxiety at work is a consequence of employee alienation. Alienation coupled with anxiety at work can act as a time bomb—firstly, affecting organizations through reduced productivity or even the failure to achieve expected objectives, and secondly, impacting employees themselves by causing feelings of boredom, vague unhappiness, dissatisfaction, and a loss of commitment and loyalty. Although much research (Sandhu and Fatima, 2021; Mahmoud et al., 2024) has treated anxiety as a prerequisite for alienation, it has also been argued that alienation is rather a necessary condition for the existence of anxiety (Kirillova et al., 2016; Vidon and Rickly, 2018). Admittedly, alienation at work has often been the subject of scientific research examining it as a mediating variable, but never, to the best of our knowledge, in the relationship linking job insecurity to the anxiety experienced by employees at work.

Some evidence has been presented to support the validity of the above findings. In fact, the results of a study carried out by Karadas et al. (2025) on a large sample of workers in five-star hotels in Northern Cyprus highlighted the mediating role of alienation at work in the relationship between emotional intelligence and the concealment of knowledge. In addition, Saudi workers in the tourism industry reported that work alienation plays a mediating role in the relationship between the effect of perceived greenwashing and non-green behavior (Elshaer et al., 2025). Furthermore, the results of a study involving employees from 92 companies operating in China demonstrated that work alienation has a mediating effect on the relationship between laissez-faire and burnout (Usman et al., 2020). Based on the above findings, the third and fourth hypotheses were developed as follows:

*H3: Alienation at work has a positive effect on employees' anxiety in hotels*.
*H4: Work alienation mediates the link between job insecurity and anxiety in hotels*.

### 2.4 Psychological safety to rectify the situation

Research into psychological safety has flourished in recent years (Frazier et al., 2017) and has consequently shown that it plays a crucial role in the workplace while generating beneficial effects for both employees and companies (Elshaer et al., 2025; Kim and Lee, 2025). Psychological safety is the extent to which employees feel that the work environment in which he or she evolves is safe for taking interpersonal risks without fear of negative outcomes (Kahn, 1990; Edmondson, 1999; Weiss et al., 2023; Lee and Seo, 2024; Shanmugaratnam et al., 2025).

First introduced by Schein and Bennis (1965), the notion of psychological safety has increasingly attracted the attention of psychology and management researchers (Edmondson and Lei, 2014). According to Gharbi et al. (2023), p. 101), in an organizational context, providing a safe environment is important to ensure positive outcomes. In simpler terms, Gharbi et al. (2023) argued that psychological safety refers to employees' feeling that they are safe from interpersonal peril, including discomfort, rejection or punishment from top management, when they make a mistake or openly express their feelings. Nguyen M. V. et al. (2025) argued that a climate of psychological safety is likely to reduce the negative impact on the mental health of workers. Moreover, Gardner and Prasad (2022) emphasized the need for a psychologically safe environment, encouraging employers to maximize psychological safety wherever possible to take full advantage of employees' authenticity in the workplace. In this respect, Liu et al. (2023) reported that psychological safety can reduce employees' feelings of anxiety. The following hypotheses were therefore formulated:

*H5: Psychological safety has a negative impact on employees' anxiety in hotels*.
*H6: Psychological safety moderates the relationship between job insecurity and anxiety in hotels*.

## 3 The conceptual model

**Figure 1:**
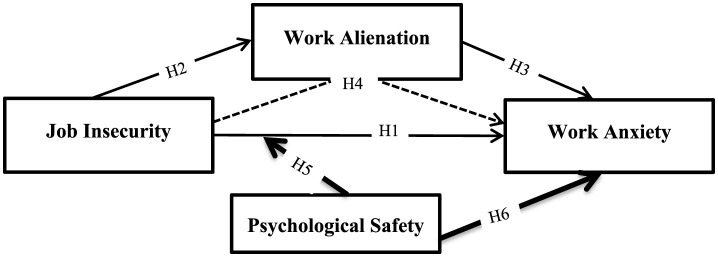
The research model.

Based on the literature review and the assumptions made above, Figure 1 shows the research model, which consisted of two sub-models. The first sub-model examined work alienation (Al) as a potential mediating variable in the relationship between job insecurity (JI) and work anxiety (Ax) experienced by hotel employees. To do this, the four steps proposed by Baron and Kenny (1986) were followed to test the veracity of the hypotheses presented in the theoretical part, particularly H1, H2, H3 and H4. The second sub-model, comprising H5 and H6, examined the impact of psychological safety (PS) on Ax and the role of PS as a moderator in the direct relationship between JI and Ax. For the statistical work on moderation, Ping's (1995) six-step procedure was followed.

## 4 Methods

### 4.1 Measurement scale

Our measurement scales were carefully selected for the research questionnaire. The research used the eight-item Karatepe (2022) to measure job insecurity (e.g., I worry that I may soon be required to work in a different location or department). Furthermore, Hamilton's (1959) four-item scale (e.g., I often have bad apprehensions) was used to measure anxiety (Ax), and Banai and Reisel's (2007) seven-item scale was used to measure alienation (Al) as a mediating variable (e.g., Often wish I were doing something else). Finally, to measure psychological safety as a moderating variable, the Edmondson (2003) was used; it includes seven items (e.g., If I make a mistake in my department, people don't really hold it against me). Appendix A 5-point Likert-type scale was also used, with questionnaire items ranging from 1 (minimum) to 5 (maximum). The means of all responses were between 1.94 and 4.52, and the standard deviations were between 0.851 and 1.502, indicating that the responses were more fairly dispersed and normally distributed (Bryman and Cramer, 2012). Several questionnaire items were arranged in such a way so as to correspond to our theoretical and practical requirements.

### 4.2 Research sample

The questionnaire forms were self-distributed to a convenience sample of 600 employees of various positions and sexes working in six luxury hotels in Sousse, Hammamet, Djerba and Tunis in Tunisia. These participants were invited to voluntarily engage in the study. The purpose of the study was explained before getting their consent for participation. A total of 516 usable forms were collected, representing a response rate of 86%. The specific characteristics of the respondents can be found in Table 1. As it could be seen, there were more female respondents (62.02%) than male respondents (37.98). The majority of the participants were married (81.40%). The years of service varied between one and over 15 years. The majority of the participants were aged 30 years or above (about 80 %). Most held a professional diploma of 2 years or higher. Almost half of the participants reported an income equivalent to 607 Euros (Table 1).

**Table 1:** Characteristics of respondents.

**Items**	**Class**	**Quantity**	**Proportion %**
Sex	Male	196	37.98
	Female	320	62.02
Marital status	Married	420	81.40
	Single	96	18.60
Length of service in the hotel	< 5 years	55	10.66
	From 5 to 10 years	104	20.16
	From 11 to 15 years	211	40.89
	More than 15 years	146	29.29
Age	< 30 years	105	20.35
	From 30 to 40 years	274	53.10
	From 41 to 50 years	119	23.06
	More than 50 years	18	3.49
Income level	Less than 607 Euros	251	48.65
	From 607 Euros to 1,212 Euros	138	26.74
	From 1,213 Euros to 1,818 Euros	108	20.93
	More than 1,818 Euros	19	3.68
Academic level	Professional 2-year diploma	265	51.36
	Bachelor's degree	27	5.23
	Bachelor's degree plus 4	204	39.53
	Bachelor's degree plus 6	20	3.88
Total		516	100%

### 4.3 Purification of scales

Principal component analysis (PCA) was conducted using the SPSS (v.25) software to test the quality of the data. For all variables measured, in this case (JI, Ax, Al and PS), the KMO indices ranged between 0.771 and 0.865. Therefore, it could be concluded that the variables lent themselves well to factorization. To assess reliability, the results showed that the Alpha values were excellent according to Nunnally (1978), (see Table 1). Moreover, the specific *p*-value for all four variables was zero, leading to the rejection of the null hypothesis.

### 4.4 Confirmatory factor analysis

The results of the CFA showed a ratio of chi-square (315) (175) *x*^2^/ddl (1.8) (Table 2). This value was acceptable as it was higher than 3. Other values, such as RMSEA = 0.039, NFI = 0.965, TLI = 0.981, IFI=0.980 and CFI = 0.985, indicated a very good model fit (Roussel et al., 2002). The results of the skewness and kurtosis coefficients confirmed that the data followed a normal distribution. To find out whether the items within our variables were intended to evaluate the same phenomenon, convergent validity was checked. This was done by examining the CR, which needed to be strictly >0.7, and the AVE. As Table 2 shows, convergent validity was confirmed (Joreskog, 1988; Jôreskog and Sorbom, 1994). Discriminant validity was also checked by reviewing the square root of the AVE, and it was confirmed (Table 2).

**Table 2:** Convergent validity.

**Variables and items**	**SL**	**CR**	**AVE**	**Mean**	**SD**	**α**
**1-Job insecurity**		0.888	0.501	0.336	0.74	0.95
JI1	0.67					
JI2	0.77					
JI3	0.64					
JI4	0.66					
JI5	0.78					
JI6	0.73					
JI7	0.71					
JI8	0.74					
**2-Work anxiety**		0.877	0.506	0.317	1.27	0.917
Ax9	0.83					
Ax10	0.86					
Ax11	0.88					
Ax12	0.89					
**3-Work alienation**		0.825	0.541	0.368	0.63	0.945
AI13	0.82					
AI14	0.83					
AI15	0.79					
AI16	0.77					
AI17	0.79					
AI18	0.72					
AI19	0.75					
**4-Psychological safety**		0.880	0.515	0.326	0.82	0.946
PS19	0.62					
PS20	0.64					
PS21	0.66					
PS22	0.78					
PS23	0.77					
PS24	0.78					
PS7	0.75					

From Table 2, it can be seen that all variables showed standardized factor loading (SFL) >0.60 (Higgins, 1998), which suggests that the variables had satisfactory reliability. Convergent validity was ensured (Higgins, 1998). As for discriminant validity, as shown in Table 2, two criteria were tested, namely the heterotrait-monotrait ratio (HTMT) and the Fornell–Larcker criterion (Leguina, 2015) (Table 3).

**Table 3:** Discriminant validity criteria.

**Variables**	**Heterotrait–Monotrait ratio (HTMT)**				**Square root values of the AVE**			
	**JI**	**Ax**	**Al**	**PS**	**JI**	**Ax**	**Al**	**PS**
JI	0.783				**0.708**			
Ax	0.633	0.776			0.521	**0.711**		
Al	0.715	0.685	0.771		0.237	0.514	**0.736**	
PS	0.626	0.641	0.610	0.765	0.518	0.612	0.237	0.718

These are the square roots of AVEs.

## 5 The results

**Figure 2:**
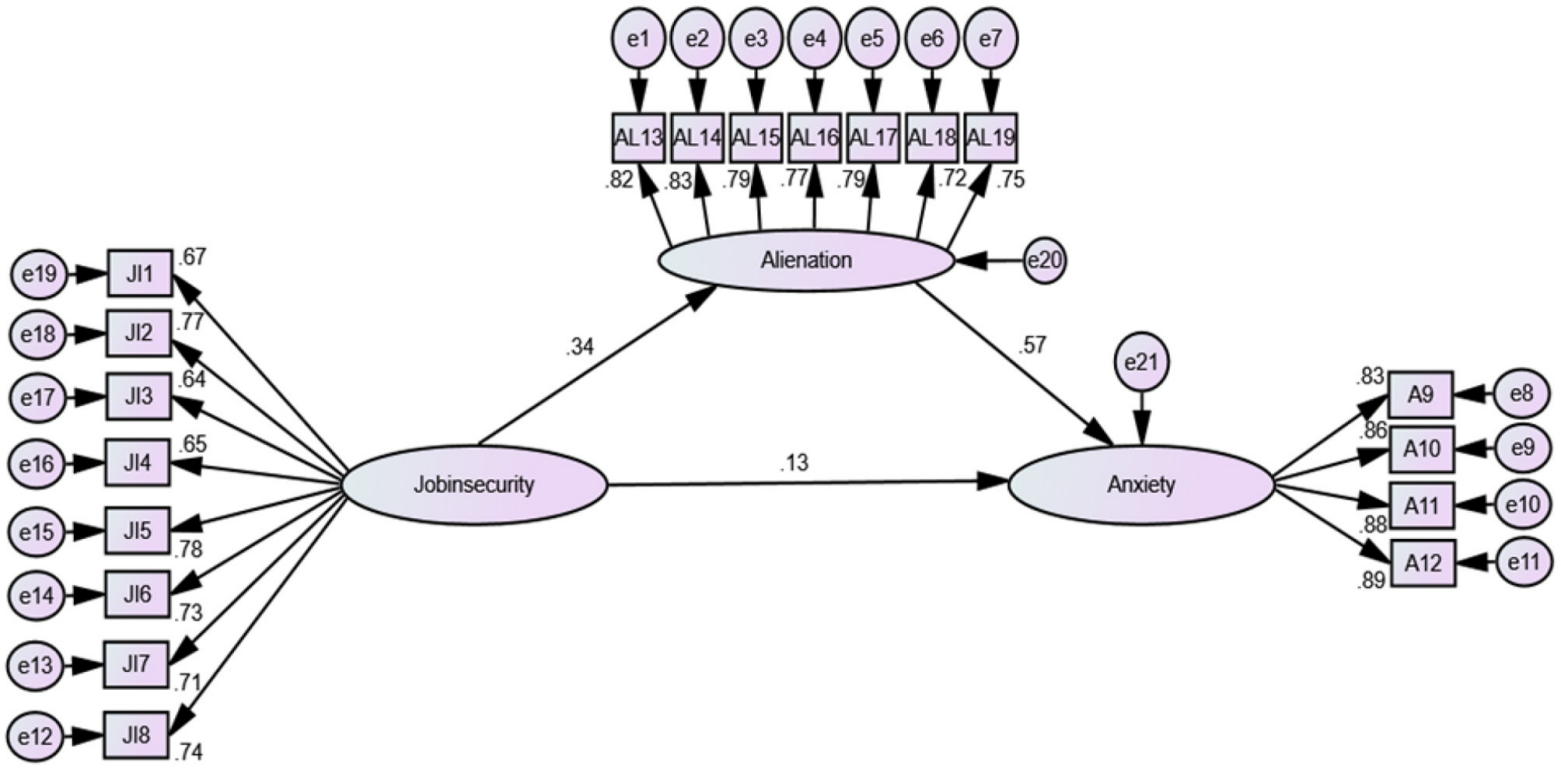
The first final sub-model.

**Figure 3:**
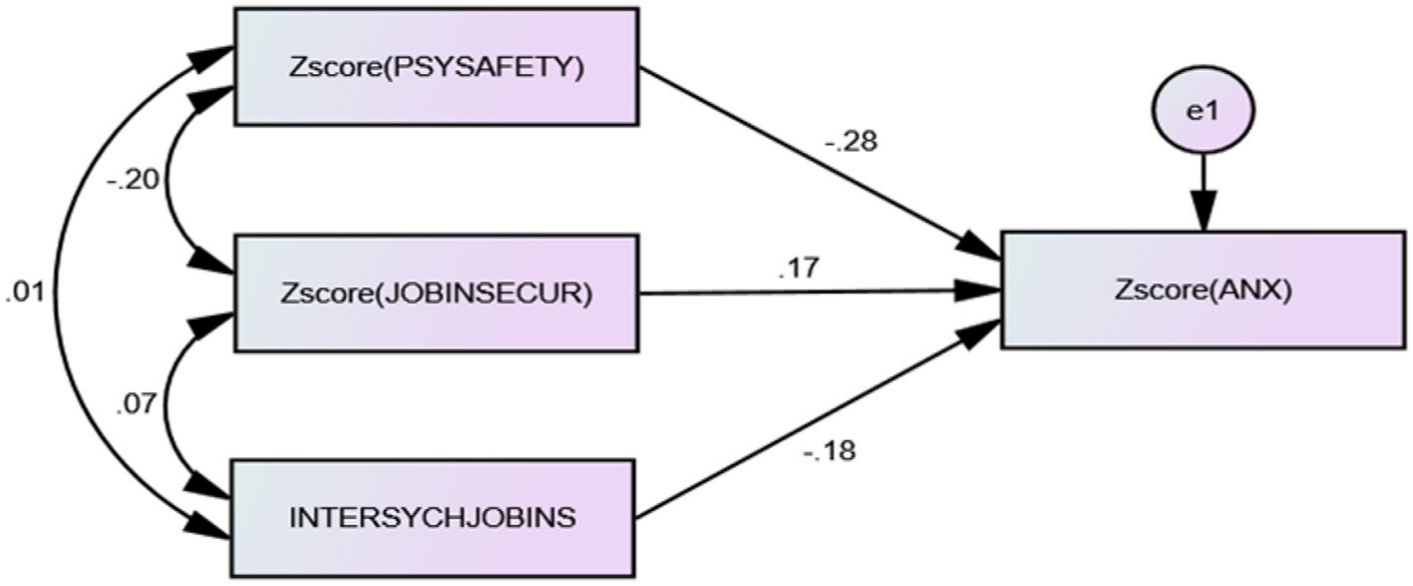
” The second final sub-model.

Having determined the reliability and validity of the latent variables in the measurement model, the following stage was to assess the internal model. The coefficient of determination (*R*^2^) was then calculated. The three variables—job insecurity, work alienation and psychological safety—together explained 51.8% of the variance in work anxiety experienced by employees. The R^2^ criterion was therefore satisfied, and according to Chin (1998), the structural model has adequate predictive capacity. All direct and indirect relationships specific to our two sub-models were examined (Table 3, Figures 2, 3) using the AMOS bootstrapping technique to evaluate the study hypotheses. All direct, mediating and moderating hypotheses were evaluated using the coefficient (β), *T*-value and *p*-value of significance.

More concretely, regarding the first sub-model (see Figure 2), JI was significantly and positively associated with Ax (β = 0.129, *p* < 0.001); therefore, H1 was accepted. It was also found that JI was significantly and positively associated with Al (β = 0.340, *p* < 0.001), thereby verifying H2. To conclude the first sub-model, Al was found to be significantly and positively associated with Ax (β = 0.569, *p* < 0.001), thereby confirming the third hypothesis.

### 5.1 The mediation test

The four steps of Baron and Kenny's (1986) approach were adopted to verify the existence of the mediation effect of Al in the relationship between JI and Ax. The first step involves verifying the relationship between JI and Ax to ensure the possibility of mediation. Indeed, the model showed that JI significantly and positively affected Ax (β = +0.129, *p* < 0.001). Next, the second step involves showing that JI significantly affects the mediating factor. The results showed that JI significantly and positively affected Al (β = +0.340, *p* < 0.001). Then, the third step involves demonstrating that the relationship between Al and Ax is significant. The results indicated that Al significantly and positively affected Ax (β = +0.569, *p* < 0.001). The fourth and final step of Baron and Kenny's (1986) approach involves verifying the partial or full mediation of Al by examining the indirect link between JI and Ax (see Table 4). Indeed, using the bootstrapping technique provided by AMOS (version 25), Table 4 shows that the relationship between JI and Ax remained significant even after the presentation of Al as a mediating variable (β = +0.321, *p* = 0.023 <0.016). Hence, Al partially mediated the relationship between JI and Ax.

**Table 4:** Result of the structural model (developed by the authors).

**Result of the structural model**	**β**	**C-R *T*-value**	**Sig**	***R* ^2^**	**Results**
H1-JI → Ax	0.129	4.176	^***^		Supported
H2-JI → Al	0.340	5.858	^***^		Supported
H3-AL → Ax	0.569	7.105	^***^		Supported
H5-Zscore PS → Ax	−0.284	−3.196	^***^		Supported
H6-Zscore (JI^*^PS) → Ax	−0.183	−2.098	0.036		Supported
Anxiety				51.8	

Model fit: χ^2^ (270, *N*= 516) = 324 (*p* < 0.001), normed χ^2^ = 1.2, GFI = 0.943, AGFI = 0.925, RMSEA = 0.020, SRMR = 0.0312, RFI = 0.981, IFI = 0.996, CFI = 0.994, TLI = 0.990, NFI = 0.981. ^***^p < 0.001.

### 5.2 The moderation test following Ping's approach

**Figure 4:**
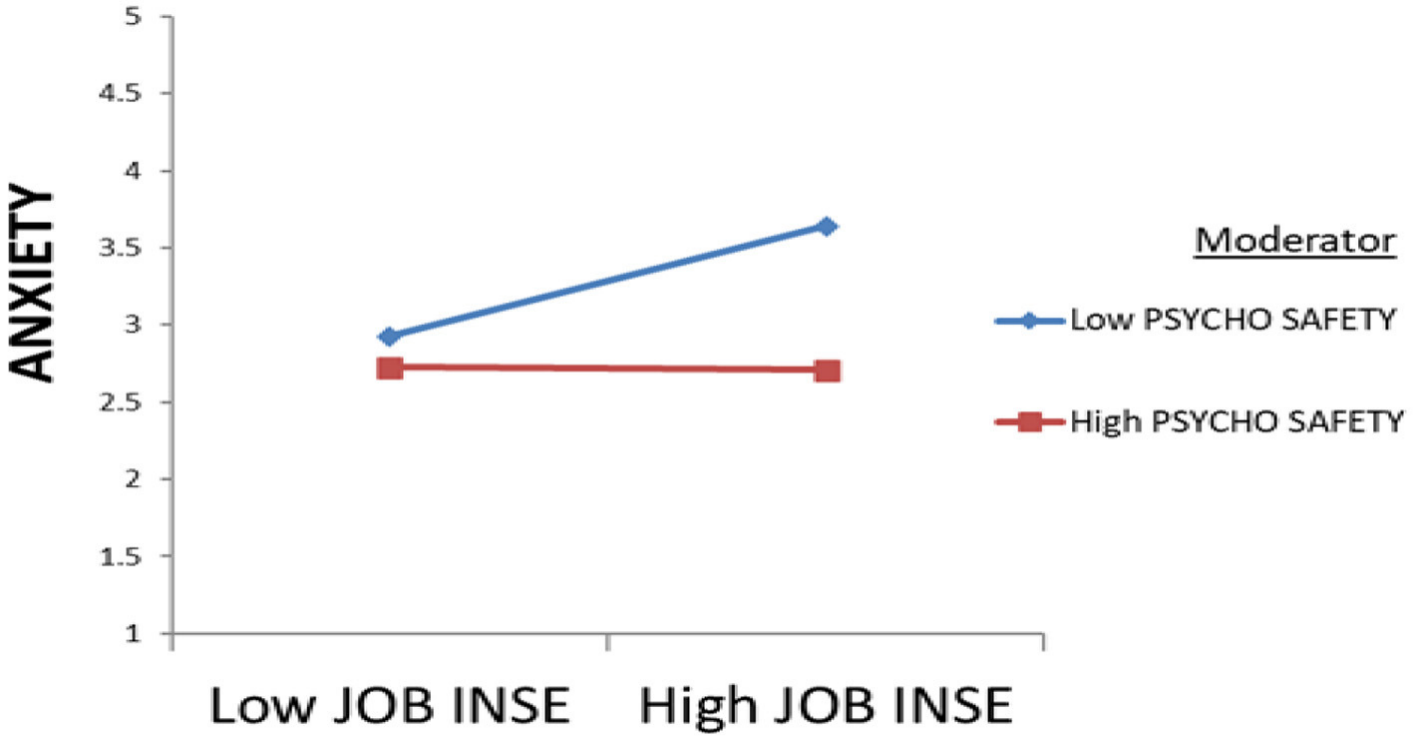
Plot for PS as a moderator on JI toward Ax.

First, Ping's (1995) approach includes checking the normal distribution of the data using skewness and kurtosis indicators. These skewness and kurtosis coefficients must be <3 in absolute value (Hair et al., 1998). In fact, the items for all variables displayed acceptable skewness and kurtosis coefficients, ranging between −3 and 3. Second, it includes checking the reliability and validity of the latent variables studied. Following a PCA and a reliability analysis, all Cronbach's alpha coefficients ranged between 0.917 and 0.950. Furthermore, the analysis of convergent and discriminant validity (see Tables 2, 3) showed satisfactory results (Fornell and Larcker, 1981). Ping (1995) insists that the reliability of the variables studied must be as high as possible, since the reliability of the moderating effect (multiplicative term: JI × PS) depends on the reliability of the two interacting variables (Aguinis, 1995). Third, to reduce multicollinearity between the multiplicative term, i.e. JI × PS, and the interacting variables, all raw data must be centered by removing their means, before calculating the multiplicative term (JI × PS) representing the moderating effect. Fourth, Ping (1995) insists on testing the interaction effect via a confirmatory factorial analysis. This analysis ensures the validity of the constructs and provides the coefficients needed to calculate the factor contribution and the error of variance of the multiplicative term (JI × PS). The results allowed all variables concerned to retain all their items, demonstrating a very acceptable quality of fit (absolute, incremental and parsimony indices; Table 4). Fifth, the approach includes calculating the interaction effect between JI and PS. This interaction is now measured by a single indicator, INTERSYCHJOBINS (Figure 3), which is simply the product of the respective sums of the indicators of JI as the independent variable and PS as the moderating variable. Finally, using standardized regression weights, the *Z*-score of JI had a significant positive association with the *Z*-score of Ax (β = 0.174, *p* = 0.006 <0.05). Furthermore, the *Z*-score of PS had a significant negative effect on the *Z*-score of Ax (β = −0.284, *p* = 0.001 <0.05). Lastly, the intercorrelation between PS and JI significantly and negatively affected the Z-score of Ax (β = −0.183, *p* = 0.036 <0.05). The moderating effect of psychological safety on the relationship between job insecurity and work anxiety was therefore confirmed in this study (see Table 5 and Figure 4). By examining the negative sign of the β coefficient linking the product JI × PS and Ax, it was evident that psychological safety reduced the positive influence of job insecurity on work anxiety. In this respect, the moderating effect was accepted (Figure 4).

**Table 5:** Type of work alienation mediation (developed by the authors).

**User-defined estimands**					**Mediation**
**Parameter**	**Est**	**Lower bounds (BC)**	**Upper bounds (BC)**	**Tow tailed significance (BC)**	
H4-JI → Al → Ax	0.321	0.110	0.665	0.016	0.016 <0.05 *PARTIAL Mediation*

## 6 Discussion

The results revealed that job insecurity has a significant positive association with work anxiety. This finding is in line with the findings reported in previous studies (Boya et al., 2008; Llosa et al., 2018 and An et al., 2023). The results confirmed that in each relationship, when an employee feels that they have fulfilled their professional obligations in the best possible way to the best of their knowledge and belief, yet the organization remains unresponsive, an unfair social exchange occurs. According to Blau (1964), this unfair social exchange can generate emotional pressure in the employee, manifesting as anger, frustration, or anxiety (Daneshvar, 2025; Haar et al., 2025).

Furthermore, the research also revealed a significant positive link between job insecurity and work alienation. This result is in agreement with the findings of Zaki and Al-Romeedy (2018), Badran and Khaled (2021) and Abouelenien et al. (2024). Indeed, our results affirm the findings of previous research (Zaki and Al-Romeedy, 2018; Badran and Khaled, 2021; Abouelenien et al., 2024), which state that one of the most important predictors of work alienation is a lack of job security. The research builds on Hobfoll's (1989) CRT, which emphasizes the deliberate desire of employees to conserve the resources that will enable them to achieve their goals. Consequently, when employees detect a threat to even one of their resources, such as their financial security, which can affect their social status, they are exposed to severe stress that risks undermining their mental and physical wellbeing. This could open a Pandora's box of unsolvable problems for both employees and their organizations, such as anxiety at work, reduced self-esteem, loss of commitment to work and alienation (Mahmoud et al., 2022).

Ultimately, the research revealed a significant positive link between alienation at work and anxiety among employees. Hence, the feeling of anxiety experienced by employees at work is dependent on the loss of meaning at work or alienation at work. This feeling of disunity emerges when employees no longer identify with the tasks they are required to perform and can no longer find fulfillment in the workplace. Consequently, a range of feelings, such as tension, anger and discomfort, linked to job performance emerges. Alienation is therefore a phenomenon recognized as part of emerging psychosocial risks, which arises when an employee no longer perceives the value or usefulness of his/her work or no longer feels concerned with the company's objectives. This ends up generating anxiety in the employee, which may have more serious repercussions if the organization fails to rectify the situation.

The empirical study highlighted two results of crucial importance. The first concerns the significant negative association between psychological safety and anxiety at work. This result confirms the findings of Kyambade et al. (2024) and Rodrigues and Figueiredo (2025), who stress the importance of psychological safety in the workplace, arguing that psychological safety makes employees less likely to experience stress or anxiety.

The second result concerns the moderating effect of psychological safety on the relationship between job insecurity and the anxiety experienced by employees in the workplace. This moderation has now been confirmed by this study (see Figure 4). Indeed, by examining the negative sign of the β coefficient linking the product (JI × PS) and Ax, it is clearly evident that psychological safety reduces the positive effect of job insecurity on anxiety at work. The analysis of the moderating effect therefore revealed that guaranteeing and promoting psychological safety in the workplace would reduce the positive impact of job insecurity on the anxiety experienced by employees in the workplace.

## 7 Implications

The research has many managerial implications. The research shows that it is the duty of decision-makers to grant their employees the opportunity to perceive organizational support, which can be an effective revitaliser of the psychological contract that binds them to their organization. In addition to psychological safety, the perception of organizational support is a concept that emerged from the work of Eisenberger et al. (1986). This initiative to concern itself with the wellbeing of its staff is proof that the organization views its employees not as a cost to be controlled but rather as a fruitful resource in which to invest. The feeling of being supported is influenced by the repetition and estimated sincerity of attitudes or signals of satisfaction and acquiescence (Blau, 1964). The signals in question may take the form of an increase in pay, promotion within the hierarchy, greater delegation of power, initiative-taking, appropriate communication, etc. These kinds of signals are perceived by the employee as support, recognition or even a reward for the efforts he or she has made for the good of the company. In this respect, Tremblay and Simard (2005, p. 108) stated that “a high perception of support may result from a conviction or belief that help will be available from the organization or its representatives when one of its members is faced with difficult or stressful situations in his or her job or personal life.”. For their part, Baydur and Uçan (2025) reported that social support in the workplace plays an important role in mitigating the negative consequences of job insecurity and preventing the negative effects on the quality of life of employees who are faced with job insecurity. In addition, based on the postulate that the way an employee is perceived can dictate his conduct—such that they may adopt passive behavior if they are regarded as passive from the outset—the theory of social exchange (Blau, 1964) states that an employee is likely to respond positively to any situation which has been favorable to him/her. This suggests that an employee who is satisfied with his or her situation will tend to take actions that benefit the organization, such as extra-role activities. Senior management is expected to show respect for its human assets by engaging in appropriate communication with each employee, offering continuous encouragement, addressing their individual problems, adopting and respecting their values and providing them with moral and financial support.

Regarding theoretical implications, the present research, quite simply, ventured onto a slippery slope. Although much research (Mannheim, 1952; Tajfel and Turner, 1986; van Dijk, 2013; Seland and Hyggen, 2021; Sandhu and Fatima, 2021; Mahmoud et al., 2024) has treated anxiety as a prerequisite for alienation, this research showed the opposite view, aligned with the work of Kirillova et al. (2016) and Vidon and Rickly (2018), which claims that alienation is rather a necessary condition for the existence of anxiety. As a result, this research brought to light new relationships, which were tested on a sample of 516 employees working in six luxury hotels in Tunisia. These findings may serve as a basis for future research, potentially enabling researchers to apply them in other contexts, both nationally and internationally. Finally, as a methodological recommendation, it stresses the fact that alienation at work has often been the subject of much scientific research (Usman et al., 2020; Karadas et al., 2025; Elshaer et al., 2025) seeking to test it as a mediating variable but never, to the best of our knowledge, in the relationship between job insecurity and the anxiety experienced by employees at work.

So, apart from being authentic, this study can be seen as a first attempt to explain that among the many causes of anxiety at work, the loss of meaning at work or alienation is a phenomenon recognized as part of the emerging psychosocial risks that surface when an individual no longer shares the company's value system, withdraws into oneself and can no longer discern the very essence of his/her work or its usefulness while feeling totally disconnected from the organization's aims.

## 8 Conclusion

The research builds upon and adds to CRT by demonstrating how job insecurity can have harmful effects on workers in hotels, particularly through the loss of meaning or alienation at work, coupled with anxiety. The mediating role of alienation in the relationship between job insecurity and anxiety helps explain the importance of psychological safety as a moderation factor, which appears in this research as a panacea for overcoming employee malaise. Indeed, when companies are no longer able to keep their promises to their employees, particularly in terms of job security, the latter may feel less motivated to perform their tasks. Tormented by a feeling of discomfort, employees, certain that they are being wronged, may develop withdrawal behaviors and, in extreme cases, be tempted to leave their companies, since their mental and physical wellbeing is being undermined. In this uncertain landscape that characterizes their daily lives, they become easy prey to the hegemony of stress, alienation at work and anxiety.

Admittedly, there are some limitations to the research, which nonetheless may pave the way for new research prospects. Firstly, the data collection was carried out using a convenience sample of employees in hotels in Tunisia. Therefore, the results could not be simply generalized despite the good sample size due to the self-reported nature of the data. Furthermore, this research did not account for the female proportion and cultural differences across Tunisian regions. Thus, future research could apply this study framework in different contexts. Furthermore, this study did not examine the role of certain demographic variables, notably sex and age, in understanding the role of work alienation in the relationship between job insecurity and work anxiety. Therefore, future studies could test the moderating role of these variables.

## Data Availability

The raw data supporting the conclusions of this article will be made available by the authors, without undue reservation.

## Appendix A

**Table A1:** The scale items.

**Variables and items**
**1. Job insecurity (Karatepe, 2022)**
My concern is whether my salary will increase
I worry that I may soon be required to work in a different location or department
My workload is probably going to become heavier in the future
I do not feel secure about the potential scope of my job
I believe my work will lose interest in the future
I am concerned that I could have a different boss in the future
I am not certain about who my coworkers will be in the near future
I do not feel secure about my prospects for advancement in my job
**2. Work anxiety (Hamilton, 1959)**
I often have bad apprehensions
Sometimes I worry
I often anticipate the worst
I have feelings of restlessness
**3. Work alienation Banai and Reisel (2007)**
Often wish I were doing something else
Facing daily tasks feels painful and boring
Time is often spent aimlessly
Feel estranged from my “real self”
Would give a good deal to live a different life
Feel all alone in the world
People are out for themselves and do not care about anyone else
**4. Psychological Safety (Edmondson, 2003)**
If I make a mistake in my department, people don't really hold it against me
Members of my department can raise difficult issues and questions
People in my department never reject others because they are different
In my department, it is safe to take risks
It is easy to ask others in my department for help
No one in my department would deliberately act in a way that undermines my efforts
In working with members of my department, my unique skills and talents are valued and used
